# Low-residue diet for colonoscopy in veterans: Risk factors for inadequate bowel preparation and patient satisfaction and compliance

**DOI:** 10.1371/journal.pone.0233346

**Published:** 2020-05-21

**Authors:** Chethan Ramprasad, Sandy Ng, Yian Zhang, Peter S. Liang

**Affiliations:** 1 Department of Medicine, NYU Langone Health, New York, New York, United States of America; 2 Division of Biostatistics, Department of Population Health and Environmental Medicine, NYU Langone Health, New York, New York, United States of America; 3 Department of Medicine, VA New York Harbor Health Care System, New York, New York, United States of America; Cleveland Clinic, UNITED STATES

## Abstract

Bowel preparation with low-residue diet (LRD) has resulted in higher patient satisfaction and similar polyp detection rates compared to conventional clear liquid diet. However, there is limited experience with LRD in veterans, in whom conditions associated with poor bowel preparation are more prevalent than the general population. To examine risk factors associated with inadequate bowel preparation, we conducted a chart review of outpatient colonoscopies at the Manhattan VA Medical Center from February 2017 to April 2018. To examine patient satisfaction and compliance, we administered an anonymous questionnaire to patients undergoing outpatient colonoscopy from March to August 2018. Patients assessed by chart review (n = 660) were 92% male with a mean age of 64 years. An adequate Boston Bowel Preparation Scale score ≥2 in each colonic segment was achieved in 94% of procedures. Higher BMI, diabetes, prior inadequate bowel preparation, bowel preparation duration of two days, and opioid use were associated with inadequate bowel preparation on univariable analysis. On multiple logistic regression, only higher BMI remained a predictor, with every one-unit increase associated with a 6% increased odds of poor bowel preparation. Questionnaire responses showed 84% of patients were willing to repeat LRD bowel preparation, 85% found the process easy or acceptable, and 78% reported full adherence to LRD. These findings demonstrate that bowel preparation quality, patient satisfaction, and compliance were all high among veterans using LRD.

## Introduction

Screening prevents deaths from colorectal cancer, but only 63% of Americans aged 50 years and older are up-to-date on screening [1]. A major barrier for individuals considering undergoing a colonoscopy is the bowel preparation process [2]. Research has shown that a low-residue diet (LRD) is a promising alternative to the conventional clear liquid diet used to prepare for colonoscopy [2, 3]. One study found that patients who consume a limited LRD before colonoscopy achieved a bowel preparation quality that was noninferior to patients on a strict clear liquid diet [4]. Furthermore, polyp detection rates, patient tolerance, and patient acceptance were similar between the two groups [4]. In other studies, patients on a LRD reported significantly higher satisfaction with bowel preparation medication, diet, and the overall preparation process [2]. Patients may also be more willing to undergo the preparation and repeat the process [3].

However, it is unclear which demographic characteristics predict inadequate bowel preparation with a LRD. Previous research has shown that older age, higher body mass index (BMI), and increased abdominal girth all independently predicted inadequate bowel preparation [5], however the reported compliance with the LRD in that study was suboptimal. Since veterans tend to be older and have a higher prevalence of medical comorbidities such as obesity that predict inadequate bowel preparation, investigating whether LRD can be used for the veteran population is of great interest [6, 7]. The efficacy of LRD in the veteran population has only been reported previously in abstract form [8].

In November 2016, a patient education pamphlet that included a LRD menu was introduced at the VA New York Harbor Health Care System (NYHHCS) Manhattan Medical Center. This replaced the previous bowel preparation instructions, which used a clear liquid diet. Compared to historical controls who underwent a clear liquid diet, those who used the LRD had a statistically non-significant increase in the proportion of individuals with adequate bowel preparation (82% vs. 86%), although the proportion with a maximum Boston Bowel Preparation Scale (BBPS) score of 9 was significantly increased (27% vs. 41%) [9]. In this study, our objectives were to identify risk factors associated with inadequate bowel preparation and to assess patient satisfaction and compliance with the LRD.

## Materials and methods

This study was approved by the Veterans Affairs New York Harbor Health Care System Institutional Review Board (#1651) and included both a chart review and questionnaire component. Prior to colonoscopy, all patients were assessed in the gastroenterology clinic and received one-on-one teaching about the bowel preparation from a registered nurse or nurse practitioner. All patients received a copy of the color educational pamphlet that formed the basis of the teaching, which included a LRD menu. The standard bowel preparation was a split-dose 2L polyethylene glycol-based solution.

To examine factors associated with inadequate bowel preparation, we conducted chart reviews on a consecutive subset of outpatient colonoscopies performed between February 2017 and April 2018 at the Manhattan VA. Informed consent was waived by the Veterans Affairs New York Harbor Health Care System Institutional Review Board. Using an automated query that was supplemented with manual data extraction, we obtained information on demographics, medical risk factors, medications, endoscopic findings, and histologic results. An adequate bowel preparation was defined as BBPS ≥2 in each colonic segment [10].

**Fig 1:**
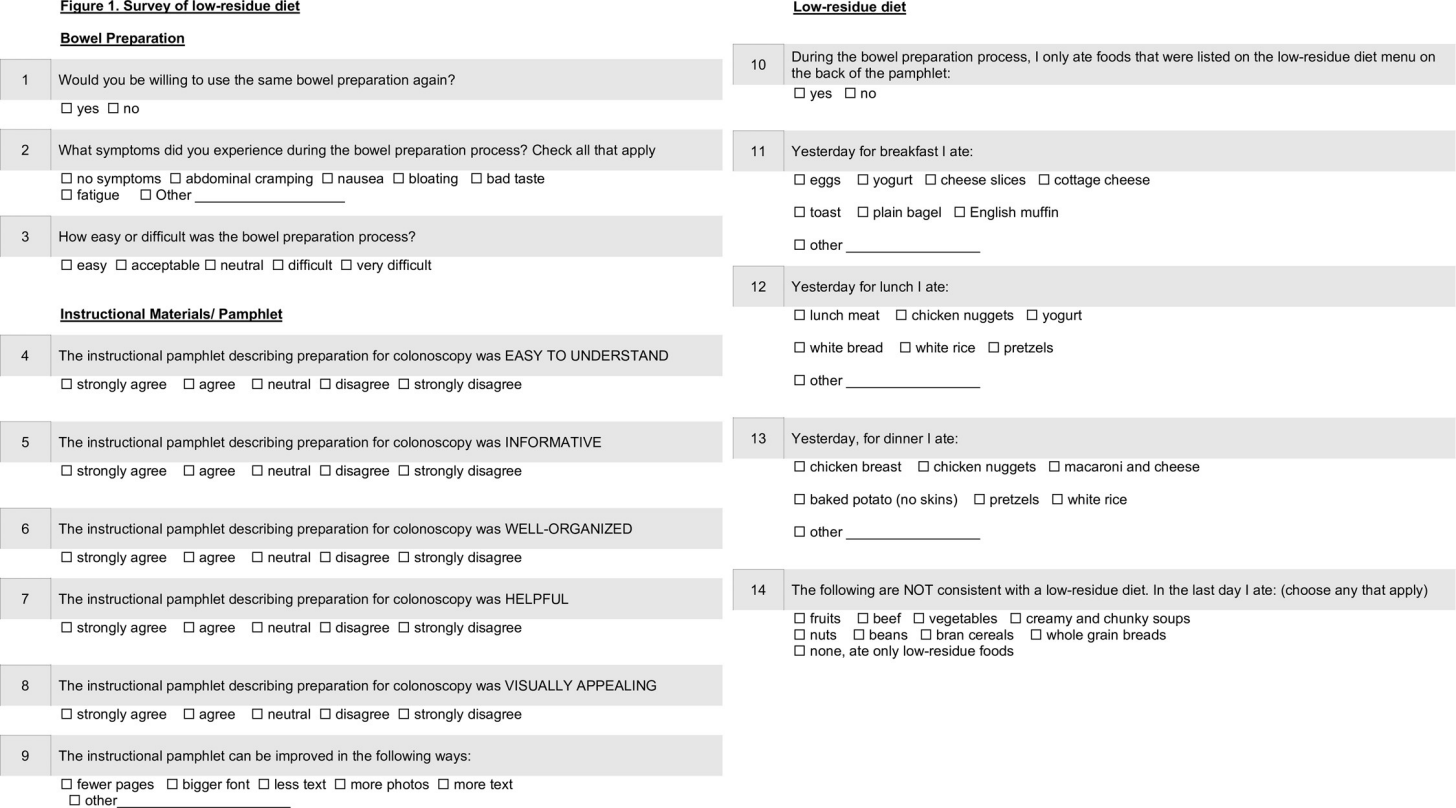
Survey of low-residue diet. Satisfaction and compliance with low residue diet were assessed with a questionnaire prior to colonoscopy. Overall, patients expressed high satisfaction with the LRD bowel preparation. Self-reported compliance with the diet was also high, with 78% of patients indicating full adherence.

To examine patient satisfaction and compliance with the LRD bowel preparation, we administered a brief, 14-item anonymous questionnaire to patients undergoing outpatient colonoscopy at our hospital from March to August 2018. As the questionnaire was primarily intended for quality improvement, its content validity was assessed internally by the study team, but pilot testing was not performed. All patients were eligible to participate. The questionnaire was optional, and consent was implied if the patient chose to participate. Separate oral or written consent was not obtained. The questionnaire addressed the following topics: 1) satisfaction with the LRD bowel preparation, 2) satisfaction with the patient education pamphlet, and 3) compliance with the LRD (Fig 1).

Statistical analyses were performed in R 3.5.2. Sample size for the analysis of risk factors associated with inadequate bowel preparation was calculated based on a baseline 15% inadequate bowel preparation in our population prior to the implementation of the educational pamphlet with LRD. Using the conventional minimum of 10 events per variable for a logistic regression model and approximately 10 variables in our model, we estimated that we would need approximately 100/0.15 = 667 patients [11]. We assessed potential predictors of inadequate bowel preparation using the chi-squared test and t-test. Variables with *P* < 0.1 on univariate analysis were entered into the multiple logistic regression model.

## Results

In the chart review portion of the study (n = 660), the vast majority of patients (92%) were men and the mean age was 64 years. Adequate bowel preparation was achieved in 94% of procedures. Table 1 shows the association between various demographic and medical factors and bowel preparation adequacy on univariate analysis. Higher BMI, diabetes, prior inadequate bowel preparation, bowel preparation duration of two days, and use of opioid medication all showed statistically significant associations with inadequate bowel preparation, whereas age, race, and smoking status did not. On multiple logistic regression, only higher BMI was statistically significantly associated with inadequate bowel preparation (OR 1.06, 95% CI 1.01–1.12, p = 0.03, Table 2). Thus, for every one unit increase in BMI, the odds of an inadequate bowel preparation increased by 6%.

**Table 1:** Association between risk factors and bowel preparation quality with low-residue diet.

Variable	Inadequate bowel preparation (n = 41)	Adequate bowel preparation (n = 619)	Univariable P-value
Age, mean (SD)	61 (10)	64 (12)	0.13
Sex, n/total (%)			0.51
Male	37/615 (6)	578/615 (94)	
Female	4/45 (9)	41/45 (91)	
Race, n/total (%)			0.22
White	11/241 (5)	230/241 (95)	
Black or African American	21/337 (6)	316/337 (94)	
Pacific Islander	0/7 (0)	7/7 (100)	
Asian	0/9 (0)	9/9 (100)	
American Indian or Native Alaskan	1/5 (20)	4/5 (80)	
Other	7/59 (12)	52/59 (88)	
Ethnicity, n/total (%)			0.19
Hispanic/Latino	11/111 (10)	100/111 (90)	
Not Hispanic/Latino	30/ 545 (6)	515/ 545 (95)	
Declined to answer	0/4 (0)	4/4 (100)	
BMI, mean (SD)	31 (6)	29 (5)	**0.02**
Smoking, n/total (%)			0.44
Current	15/150 (10)	135/150 (90)	
Non-smoker	13/187 (7)	174/187 (93)	
Former	6/149 (4)	143/149 (96)	
Diabetes, n/total (%)			**0.03**
Yes	17/171 (10)	154/171 (90)	
No	24/489 (5)	465/489 (95)	
Constipation, n/total (%)			0.86
Yes	9/130 (7)	121/130 (93)	
No	32/530 (6)	498/530 (94)	
Dementia, n/total (%)			0.14
Yes	2/8 (25)	6/8 (75)	
No	39/652 (6)	613/652 (94)	
Cirrhosis, n/total (%)			0.15
Yes	3/22 (14)	19/22 (86)	
No	38/638 (6)	600/638 (94)	
Prior inadequate bowel prep, n/total (%)			**0.04**
Yes	10/84 (12)	74/84 (88)	
No	31/576 (5)	545/576 (95)	
Bowel prep duration, n/total (%)			**<0.01**
One day	10/164 (6)	154/164 (94)	
Two day	8/85 (9)	77/85 (91)	
Opioid use, n/total (%)			**0.01**
Yes	6/34 (18)	28/34 (82)	
No	35/626 (6)	591/626 (94)	

Abbreviations: SD, standard deviation; BMI, body mass index

**Table 2:** Risk factors for inadequate bowel preparation on multiple logistic regression.

Variable	OR (95% CI)	P-value
BMI, per unit increase	**1.06 (1.01,1.12)**	**0.03**
Diabetes	1.70 (0.83,3.48)	0.14
Prior inadequate bowel prep	1.60 (0.67,3.81)	0.29
Two day bowel prep	2.01 (0.95,4.25)	0.07
Opioid use	2.31 (0.76,6.98)	0.14

Abbreviations: OR, odds ratio; CI, confidence interval; BMI, body mass index

The results of the questionnaire portion of the study (n = 251) are shown in Table 3. Overall, patients expressed high satisfaction with the LRD bowel preparation. Eighty-four percent of patients were willing to repeat the bowel preparation, 85% found the process easy or acceptable, and 44% did not experience any symptoms during the preparation process. Self-reported compliance with the diet was also high, with 78% of patients indicating full adherence. The most commonly consumed LRD foods included the following: breakfast eggs (55%), lunch meat (35%), chicken breast for dinner (32%), white bread for lunch (27%), baked potato for dinner (15%), and white rice for dinner (14%). Of the patients who were not compliant with the diet, the most commonly eaten food items were fruits (5%), beef (5%), and creamy chunky soups (3%). The most common symptoms experienced with bowel preparation were bad taste (20%), abdominal cramping (17%), and bloating (17%).

**Table 3:** Results of patient questionnaire on LRD bowel preparation (n = 244).

Question	n (%)
Satisfaction with LRD bowel preparation	
Willing to repeat bowel preparation	204 (84.1%)
Found bowel prep easy or acceptable	191 (85.2%)
Had no symptoms with bowel prep	108 (44.2%)
Satisfaction with instructional pamphlet	
Easy to understand	199 (89.2%)
Informative	202 (90.0%)
Well organized	200 (86.9%)
Helpful	205 (91.2%)
Visually appealing	166 (66.5%)
Self-reported compliance with LRD	
Only ate LRD	190 (78.1%)

## Discussion

In this single-center study of veterans undergoing bowel preparation with a LRD, we found 94% adequate bowel preparation as well as high levels of patient-reported satisfaction and compliance. Since veterans have a higher prevalence of obesity and smoking than the general population [6, 7], and these comorbidities have been previously associated with poor bowel preparation, our results suggest LRD can be successfully implemented even in older patients with multiple comorbidities.

We found that higher BMI was the only independent predictor of poor bowel preparation with LRD. Existing literature examining the relationship between BMI and bowel preparation quality have shown conflicting results. Our findings are consistent with other retrospective studies that suggest overweight or obese individuals are more likely to have suboptimal bowel preparation. Borg et al found that individuals with BMI ≥25 kg/m^2^ were more likely to have inadequate bowel preparation [12], and Fayad et al came to a similar conclusion for persons with BMI ≥30 kg/m^2^ using split-dose bowel preparation [13]. Both studies used the Aronchick scale, which is a qualitative scoring system that may be subject to greater interobserver variability than the quantitative BBPS score. On the other hand, a recent large prospective study of 1314 patients that used a split-dose regimen and the modified Aronchick scale did not identify a correlation between higher BMI and bowel preparation [14]. A smaller prospective study of 99 patients that used a low-volume sodium picosulfate preparation and the BBPS score found similar results [15]. Our study is the first to identify obesity as a predictor for poor bowel preparation using a LRD. The discrepancy in published results may be attributable to differences in the bowel preparation regimens as well as instructions provided to study participants. Given that obesity is an established risk factor for colorectal cancer and colonoscopy with adequate bowel preparation protects against colorectal cancer, additional research to define this relationship is needed.

A number of other factors—including age, smoking, diabetes, and opioid use—have been previously identified as predictors of inadequate bowel preparation using a clear liquid diet [12–14, 16]. That these factors were not identified as significant predictors is likely due to several limitations of our study. First, most patients in our study were above the age of 60, which restricted the spectrum of age for the analysis and could have affected the results. Second, because we used administrative codes to extract clinical information, missing or incorrectly coded data may have influenced the findings. Third, the number of patients with poor bowel preparation was smaller than anticipated. Therefore, we were slightly underpowered to evaluate all the variables of interest in the regression model. However, the small proportion of inadequate bowel preparation observed supports the clinical utility of the LRD. A fourth limitation is that the patient satisfaction portion of the questionnaire relate to a bowel preparation process that includes the LRD rather than the LRD in of itself. We chose to ask a single question about bowel preparation in order to minimize questionnaire length and maximize clinically relevant responses, since the diet is an integral component of the bowel preparation process.

The effectiveness of colonoscopy is dependent on high-quality bowel preparation, which in turn depends on patient comprehension and adherence to the preparation process. Most patients in our study were satisfied with our educational pamphlet and LRD overall, although the visual appeal of the pamphlet scored poorly and improving the design may boost future adherence. Many educational interventions have been trialed to improve preparation quality, including pamphlets, videos, and telephone calls. A systematic review found that the vast majority of these interventions led to statistically significant improvements in bowel preparation, but since most were conducted at single centers generalizability to other settings may be limited [17]. Although our study was also conducted at a single center, it is part of the largest integrated healthcare system in the US and therefore may be more applicable to the nine million veterans enrolled in the VA each year. Nevertheless, more research is needed to determine the most effective patient education approach, and the optimal method will likely vary based on local patient demographics and availability of resources.

## Conclusion

Our study provides further evidence that a bowel preparation using LRD is effective and well-accepted, even in a population of older veterans with multiple comorbidities. The relationship between higher BMI and poor bowel preparation, if confirmed, may warrant targeted interventions for the increasing number of overweight and obese individuals. Patients reported high satisfaction and compliance with the LRD, which was introduced as part of an educational pamphlet. Future work is needed to improve the visual appeal of the educational pamphlet and further emphasize the importance of adhering to the LRD.
